# The pervasive relevance of COVID-19 within routine paediatric palliative care consultations during the pandemic: A conversation analytic study

**DOI:** 10.1177/0269216320950089

**Published:** 2020-08-16

**Authors:** Katie Ekberg, Lara Weinglass, Stuart Ekberg, Susan Danby, Anthony Herbert

**Affiliations:** 1 School of Early Childhood and Inclusive Education, Queensland University of Technology, Australia; 2 School of Psychology & Counselling, Queensland University of Technology, Australia; 3 Centre for Healthcare Transformation, Queensland University of Technology, Australia; 4 Australian Research Council Centre of Excellence for the Digital Child, Queensland University of Technology, Australia; 5 Children’s Health Queensland Hospital and Health Service, Australia; 6 Centre for Children’s Health Research, Australia; 7 School of Nursing, Queensland University of Technology, Australia

**Keywords:** Palliative care, COVID-19, pandemics, communication, child

## Abstract

**Background:**

The importance of caring for children with complex and serious conditions means that paediatric palliative care must continue during pandemics. The recent pandemic of Coronavirus Disease 2019 (COVID-19) provides a natural experiment to study health communication during pandemic times. However, it is unknown how communication within consultations might change during pandemics.

**Aim:**

This study, a sub-study of a larger project, aimed to examine real-world instances of communication in paediatric palliative care consultations prior to and during the COVID-19 pandemic to understand how clinicians and families talk about the pandemic.

**Design:**

Paediatric palliative care consultations prior to, during, and immediately following the initial peak of COVID-19 cases in Australia were video recorded and analysed using Conversation Analysis methods.

**Setting/participants:**

Twenty-five paediatric palliative care consultations (including face-to-face outpatient, telehealth outpatient and inpatient consultations) were video recorded within a public children’s hospital in Australia. Participants included 14 health professionals, 15 child patients, 23 adult family members and 5 child siblings.

**Results:**

There was a pervasive relevance of both serious and non-serious talk about COVID-19 within the consultations recorded during the pandemic. Topics typical of a standard paediatric palliative care consultation often led to discussion of the pandemic. Clinicians (55%) and parents (45%) initiated talk about the pandemic.

**Conclusions:**

Clinicians should not be surprised by the pervasiveness of COVID-19 or other pandemic talk within standard paediatric palliative care consultations. This awareness will enable clinicians to flexibly address family needs and concerns about pandemic-related matters that may impact health and wellbeing.

**What is already known about the topic?**

- The urgency of caring for children with complex and serious conditions ensures that care must continue during the Coronavirus Disease 2019 (COVID-19) pandemic.
- As yet, guidelines for communication with families about the COVID-19 pandemic are not based on direct observational evidence of actual communication practices within palliative care during the pandemic.

**What this paper adds?**

- The current study provides evidence of the pervasive relevance of communication about the COVID-19 pandemic during clinician-family paediatric palliative care consultations.
- There was a pervasive relevance of serious and non-serious talk about the pandemic.
- Topics typical of standard paediatric palliative care consultations often led to discussion of the pandemic, including medical discussions and psychosocial and lifestyle discussions.
- Clinicians (55%) and parents (45%) initiated talk about the pandemic.

**Implications for practice, theory, or policy**

- Clinicians should expect and be prepared for the pervasiveness of talk about the COVID-19 pandemic within standard paediatric palliative care consultations, so that they can be flexible in how they respond to families.
- Future guidelines should consider the pervasive and varied ways that conversations about a pandemic are raised within and across routine consultations.

## Introduction

The Coronavirus Disease 2019 (COVID-19) has had a significant impact on the global human population since its development into a pandemic in early 2020. This paper examines care of children with life-limiting conditions during the COVID-19 pandemic. Although children, overall, appear to be less affected than adults,^1–4^ the implications of COVID-19 for children with life-limiting conditions is less clear. The lower incidence rate of the disease amongst children, and an apparently low rate of severe cases, means little was known during the early months of the pandemic about the potential impact of COVID-19 on children.^5–8^ There was some evidence that children with severe COVID-19 had pre-existing comorbidities,^9,10^ although this only emerged many months into the pandemic. In addition, children were potentially vulnerable to both health and psychosocial^11^ implications of the pandemic given the unprecedented changes to their normal daily routines (e.g., school closures, changes to parents’ employment, and cancellations of social and sporting commitments).^8^

Communication is pivotal for realising the holistic focus of palliative care,^12–14^ providing the means to understand the physical, mental, social, cultural, and spiritual needs of patients and their families.^15–17^ The urgency of caring for children with complex and serious conditions requires that care must continue during the pandemic, albeit with adjustments to service delivery.^18,19^ To reduce the risk of infection, a greater proportion of palliative care consultations during the pandemic were reconfigured to telehealth.^18–20^ Beyond changes to the *medium* for consultations, it is still relatively unknown how the *content* of consultations within paediatric palliative care also might have changed during the pandemic. Guidelines and scripts were developed to guide clinicians confronted with the rapid changes of the pandemic, both within healthcare systems and across societies more generally.^21–23^ Initial resources about communication during the pandemic focused on communicating with and about patients who had COVID-19.^24,25^ Additional resources were developed later for communicating with patients and families receiving standard, ongoing care for other conditions during the pandemic.^26^ These guidelines provided suggestions for how clinicians might set up a specific conversation about the pandemic with patients as part of an otherwise routine clinical encounter. These guidelines were not, however, based on direct observational evidence of communication within palliative care during the COVID-19 pandemic. Moreover, most guidance focussed on adult, rather than paediatric, care. The current study addresses these gaps, by comparing video-recordings of actual, real-world instances of communication in paediatric palliative care consultations prior to and during the COVID-19 pandemic. The peak months of the pandemic became an opportunity to understand how the provision of paediatric palliative care is maintained, potentially with adaptations, during the uncertainty of a pandemic.

## Method

### Setting

**Figure 1.**
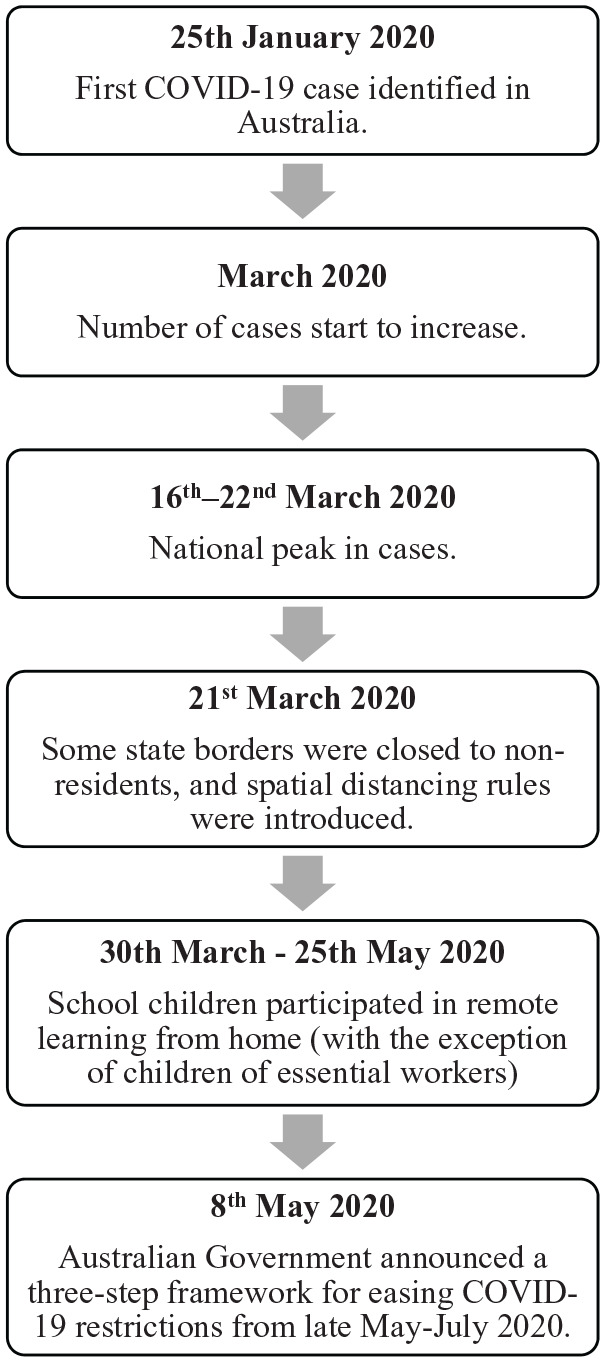
The epidemiological trajectory of COVID-19 in Australia March-May 2020.

This study was part of a larger project that examined communication between healthcare professionals and families within paediatric palliative care services in Australia. Analysis was based on a corpus of 25 paediatric palliative care consultations that were video recorded in a public children’s hospital in Australia. Fifteen of these consultations were recorded in the months of September 2019–February 2020, prior to COVID-19 being declared a pandemic.^27^ Ten consultations were recorded during the months of March–May 2020, which was during and immediately following the initial peak of COVID-19 cases in Australia. The epidemiological trajectory of the initial peak of COVID-19 in Australia can be found in Figure 1.^28^

### Data collection

Staff and families involved in the paediatric palliative care service were informed about the project by a member of hospital staff. All participants provided informed written consent themselves (adults) or by their guardian (for all child patients). Prior to the start of each recorded consultation, a clinician set up two video cameras in the consultation room. In the telehealth consultations, one camera captured the interactions of the clinicians in the room and the other camera was directed towards the computer showing the family via videoconference. No researchers were present during the video-recorded consultations.

### Data analysis

The video-recorded data were transcribed using the standard conversation analytic transcription conventions developed by Jefferson (see Appendix 1 for transcription notations).^29^ The transcripts include details of pauses, overlapping talk, intonational contours and non-verbal communication found to be consequential for how participants manage social interactions. All names of people and places in the transcripts are pseudonyms. The data were analysed using conversation analysis (CA) by authors KE, LW, SE, and SD.^30^ Increasingly, the method of conversation analysis is used in clinical settings,^31,32^ including in paediatric palliative care, to capture the complexity of the interactions as they unfold, moment-by-moment.^33,34^

A CA approach uses observation to ensure analysis is based on participants’ experiences and differs from other research approaches that begin with assumptions, intuitions, or hypotheses.^35^ Analysis involves building an exhaustive collection of sequences of interaction that identify a particular communication practice, and then undertakes a turn-by-turn examination of each and all of these sequences to understand how specific conversational practices influence an ongoing interaction.^30^ In this analysis, all sequences of talk related to COVID-19 and the associated changes to lifestyle during the pandemic were extracted from the appointments recorded during the pandemic (*n* = 33 fragments) and were examined for how this talk was initiated and responded to within the broader sequence of conversation within the consultation. These fragments were also compared with similar sequences of interaction in the pre-pandemic data. Fragments that display clear examples of the phenomena being discussed are presented below. In this paper, we have included examples of both serious and non-serious pandemic talk to show the breadth of the types of sequences identified in the corpus.

### Ethical approval

This study was approved by the Children’s Health Services Queensland Human Research Ethics Committee (HREC/18/QRCH/86) and Queensland University of Technology Human Research Ethics Committee (1800000468), in addition to site-specific governance approvals. The study adhered to the principles of the Australian National Health and Medical Research Statement on Research Involving Human Subjects.

## Analysis

Key demographic information about each appointment is found in Table 1. The pre-pandemic consultations comprised 13 face-to-face outpatient consultations and two face-to-face inpatient consultations. The pandemic consultations comprised four telehealth outpatient consultations and six face-to-face inpatient consultations.

**Table 1.** Appointment demographics.

Appointment number	Appointment type	Child in appointment (Y/N)	Family members in appointment	Healthcare professionals in appointment
**Pre-pandemic**				
S1_F09_E01_2019-08-22	F2F outpatient	Y	Mother	Doctor
S1_F10_E01_2019-09-05	F2F outpatient	Y	Mother	Doctors ×2
S1_F11_E01_2019-09-19	F2F outpatient	Y	Mother Older sister	Doctors ×2
S1_F12_E01_2019-11-19	F2F outpatient	Y	Mother Father Older sister	Doctor Training Nurse
S1_F12_E02_2020-02-10	F2F outpatient	N	Mother Father	Doctor
S1_F13_E01_2019-11-26	F2F outpatient	Y	Mother Father	Doctor Medical student Physiotherapist
S1_F14_E01_2019-11-28	F2F outpatient	Y	Father Younger brother	Doctor
S1_F14_E02_2020-02-20	F2F outpatient	Y	Father Support worker	Doctor Nurse
S1_F15_E01_2019-12-12	F2F outpatient	Y	Mother Twin younger brothers	Doctor Medical student
S1_F17_E01_2019-12-17	F2F outpatient	Y	Mother Father	Doctor
S1_F18_E01_2020-02-10	F2F inpatient	Y	Mother	Doctor Nurse
S1_F18_E02_2020-03-13	F2F inpatient	Y	Mother	Doctor
S1_F19_E01_2020-02-20	F2F outpatient	N	Mother Father	Doctor
S1_F20_E01_2020-02-24	F2F outpatient	Y	Mother Support worker	Doctor Registrar Training nurse
S1_F21_E01-2020-02-27	F2F outpatient	Y	Mother	Doctor Nurse
**During initial peak of pandemic**				
S1_F10_E02_2020-04-16	F2F inpatient	Y	Mother	Doctor Nurse
S1_F10_E03_2020-04-29	F2F inpatient	Y	Mother	Doctor Nurse
S1_F12_E03_2020-05-08	Telehealth	Y	Mother Father	Doctor
S1_F17_E02_2020-05-07	Telehealth	N	Mother	Doctor Nurse Medical student
S1_F23_E01_2020-04-09	Telehealth	Y	Father Twin sister	Doctor Fellow
S1_F24_E01_2020-04-16	F2F inpatient	Y	Mother	Doctor Nurses ×2 Music Therapist
S1_F24_E02_2020-04-17	F2F inpatient	Y	Mother	Doctor Nurse
S1_F24_E03_2020-04-22	F2F inpatient	Y	Mother	Doctor Nurse
S1_F24_E04_2020-05-19	F2F inpatient	Y	Mother	Doctor Nurses ×3
S1_F25_E01_2020-04-30	Telehealth	Y	Mother Father Younger sister	Doctor

F2F: face-to-face consultation.

Consultations had a mean duration of 41 ½ min (SD = 23.3), with a total of 17 h and 15 min of data. Participants included 14 health professionals (including doctors, nurses, and allied health professionals), 15 child patients, 23 adult family members, and 5 child siblings present in the consultations. Six families had two or more consultations recorded. Key demographics of the child patients are presented in Table 2. The children ranged in age from 3 to 15 years old (M = 7.73 years, SD = 4.38) and sixty percent of the patients were male. No children in the study had received a diagnosis of Coronavirus at the time of their recorded consultation(s). Child diagnoses included Neurological (*n* = 6), cerebral palsy (*n* = 4), metabolic (*n* = 3), genetic (*n* = 1), and rare (*n* = 1).

**Table 2.** Child patient demographics.

Participant	Sex	Age	Diagnosis
F09	F	6	Cerebral Palsy
F10	M	5	Pontocerebellar Hypoplasia
F11	F	14	Cerebral Palsy
F12	M	7	Sanfillippo Syndrome (mucopolysaccharidosis type III)
F13	M	15	Sanfillippo Syndrome (mucopolysaccharidosis type III)
F14	F	9	Cerebral Palsy
F15	M	3	Epileptic Encephalopathy
F17	M	3	Phelan Mcdermid Syndrome
F18	M	3	Epileptic Encephalopathy
F19	F	13	Cerebral Palsy
F20	F	14	Epileptic Encephalopathy
F21	M	7	Lysosomal Storage Disorder
F23	M	9	Lymphangiomatosis
F24	F	4	Pontocerebellar Hypoplasia
F25	M	4	Genetic Neurodevelopmental Disorder

Analysis of routine care consultations recorded during the COVID-19 pandemic revealed the pervasive relevance of the pandemic during consultations. Topics typical of standard paediatric palliative care consultations often led to discussion of the pandemic. This was introduced between one and seven times within each of the ten consultations recorded during the pandemic (total = 33, mean = 3.3 times per consultation) and often led to extended sequences of interaction. Within these consultations, topics typical of standard paediatric palliative care consultations often led to discussion of the pandemic. These topics were diverse, spanning medical discussions (e.g. pain medication) and psychosocial and lifestyle discussions that are a key part of the holistic, supportive care provided within palliative care services (e.g. school, parents’ caring/work responsibilities). Of the 33 fragments analysed, the pandemic was raised by clinicians in 18 fragments (55%) and parents in 15 fragments (45%). The pervasive and varied nature of the talk about the pandemic is analysed in detail in the below fragments, which are exemplars of the broader corpus of consultations. First, some fragments of typical talk from consultations recorded prior to the pandemic are considered. These will be compared with similar fragments from the consultations recorded during the pandemic.

### Consultations prior to the COVID-19 pandemic

Fragments 1-4, recorded prior to the peak of the pandemic, show examples of typical sequences of conversation within palliative care consultations before the pandemic. Each fragment involves a suggestion or enquiry from the doctor often observed within these types of consultations: making a suggestion about pain medication (Fragment 1); enquiring about the child’s school (Fragment 2); enquiring about the parent’s work responsibilities (Fragment 3); and enquiring about what the child is watching on a digital tablet (Fragment 4).

**(1)  [S1_F12_E02_2020-02-10 48:41]**

01   (12.1)

02 Doc: U::m (3.0) so the Panadol um he was on three seventy five

03   but he could probably ah go up to four fifty now.

04 Dad: °Mmhm°.

05   (2.0)

06 Doc: And Nurofen he could go up to 300 °milligrams°.

07   (21.0)

08 Doc: Oh so um Hannah says she can do a phone order so that’s good.

09  (7.0)

**(2) [S1_F11_E01_2019-09-19 23:20]**

01 Doc: Which school are you- is she at?

02 Mum: Magpie Park [near Flagstaff]

03 Doc:  [ Magpie Park ]

04 Doc: Okay yeah an’ is she (.) what level is she sort’ve at school

05   sort’ve (0.5) um I guess at the upper [levels maybe,]

06 Mum:      [ Oh:::::: ]

07 Doc: or sort’ve high school at least?

08 Mum: Oh [yeah. Yep]

09 Doc:  [Yeah yep] yep mid-high school I guess [they’d call it? ]

10 Mum:            [Something like that.]

11   I suppose.

12 Doc: Yeah okay.

13 Mum: Yeah she’s a senior.

14 Doc: Yeah.

15   (2.1)

16 Doc: U::m so then in terms of practical supports you um you’ve

17   mentioned the (0.3) belt particularly for plane flights. . .

**(3) [S1_F14_E01_2019-11-28 36:16]**

01 Doc: And u- u- m- it sounds like you’ve got a full-time job looking

02  after Bianca? Yeah,

03  (0.9)

04 Dad: Um what do you mean?=

05 Doc: =Like do you work as w↑ell [or yeah okay]

06 Dad:         [Yeah I- so I] started a business.

07 Doc: Okay wow yeah [what’s] your business?

08 Dad:      [Yeah. ]

09 Dad: Uh lawn mowing an’ that.

10 Doc: Okay.

11 Dad: Yep.

**(4) [S1_F17_E01_2019-12-17 06:52]**

01 Doc: heh he:h what- what’s he watching?

02 Dad: Oh he’s watching a car one.

03 Doc: Yeah.

04 Mum: Just Blippi it’s only [thing] he watches.

05 Doc:      [Okay ]

06 Doc: Yeah yeah what’s it called Blippi?

07 Dad: [Blippi]

08 Mum: [Blippi] it’s a- it’s an American education: guy

09 Doc: [Okay]

10 Mum: [that] goes ar[ound] showing them different things

11 Doc:  [okay]

12 Mum: [like cars or] [£rasp]berry farms£ or

13 Doc: [yeah heh heh][ yeah]

14 Doc: Yeah yeah

15 Mum: I don’t know why he likes it but he does.

16 Doc: Yeah.

These typical sequences of conversation also were observed in consultations recorded during the pandemic. As will be shown in the next section, however, the sequences shifted to a different trajectory relevant to life during the pandemic. A comparable instance to each of the above fragments is considered now, drawn from consultations recorded during the pandemic.

### Consultations during the COVID-19 pandemic

The next five fragments recorded during the COVID-19 pandemic demonstrate how similar topics of conversation were initiated, and how they soon shifted to talk about COVID-19 by either clinician or parent. In the first example below, a concern was raised by a parent about the treatment of their child during the pandemic period. In the initial conversation, a telehealth consultation between the doctor and father, the father had mentioned that ibuprofen seemed to work better than paracetamol for relieving their child’s headaches. This topic of pain relief has been raised again now, this time by the doctor (lines 1-2), who recommends giving the child *Nurofen®* (a popular brand of ibuprofen) for pain relief from these headaches.

**(5) [S1_F23_E01_2020-04-09 13:49]**

01 Doc: Okay yeah no >that’s really good< and maybe .hh try n’ sort’ve

02   give him medications: ea- like the Nurofen early maybe,

03  (1.0)

04 Dad: Yeah [yeah] but we’ve had some concerns with corona[virus]

05 Doc:  [yeah]           [°Mm° ]

06 Dad: around at the moment and (.) y’know there is some (0.4) sort’ve

07   murmurs out there that it’s: it’s not the best thing to use

08   Nurofen at the moment but um y’know a lot of the times we don’t

09   really have any choice.

10 Doc: Yeah I have a- yeah I di- I have looked into the coronavirus and

11   the Nurofen, (.) u::m (.) and um (.) the- (.) the- (.) there was a

12   suspicion that maybe (.) u::m some patients who had coronavirus

13  (.) uh there was an association with Nurofen and poorer outcomes

14  [but] first >thing< is that these were much older patients,

15 Dad: [Yep]

16 Doc: and many of them sorta [had] things like heart conditions and

17 Dad:       [Yep]

18 Doc: lung- and lung conditions and renal issues,

19   (0.2)

20 Doc: um and it was ONLY the patients with coronavirus that had this

21   ↑issue,

22   (0.4)

23 Doc: um so- so- and the World Health Organisation have actually come

24   out and said that they think (.) um that (.) um taking Nurofen is

25   okay with (.) coronavirus, so just to reas[sure] you,

26 Dad:            [Okay]

27 Doc: u:m but ↑if- if- >and I guess hopefully< Sam- Sammy doesn’t

28   sort’ve have any issues with um (0.2) getting coronavirus but um

29   (0.4) um

30 Dad: We’ve been £locked down fo(h)r a few weeks [now ]£

31 Doc:            [Yeah] yeah and um (.)

32   we seem to be doing okay in ((State)) at the moment although we

33   don’t want to sort of be too um confident but (.) but maybe um

34   it’s OKAY while he’s well to take it, but maybe if he did get sick

35   you might stop it then, yeah so um so yeah.

36   (0.5)

37 Doc: Does that reassure you?

38   (1.5)

39 Dad: Yeah.

40 Doc: Yeah

41   (.)

42 Doc: [So-]

43 Dad: [Yep] that’s fine.

44 Doc: And the World Health Organization=>they actually put out a< and I

45   can try to send you the link but they actually put out a sort of a

46   statement saying that there’s no real association between Nurofen

47   and- and um worse outcomes with coronavirus.

48   (1.0)

49 Dad: Okay.

50 Doc: Mm.

51 Dad: That’s good to know.

52 Doc: Yeah.

53   (0.5)

54 Doc: The ↑other thing that’s a little bit reassuring is- and it’s not

55   totally reassuring but y’know that (0.2) w- they’re tending to

56   find that children are having less severe (0.3) and the length of

57   their illness is shorter as well and it’s really seems to be an

58  ↑adult illness,

59   (.)

60 Doc: U::m (.) [but] I know- I know you know patients with a complex

61 Dad:   [Yep]

62 Doc: illness such- such as Sammy y’know he: y’know you also need to (.)

63   u:m .tch y’know look after Sammy as well and protect him as well,

64   so yeah but there is a little bit of a [reas]surance but I’m also

65 Dad:        [Yes ]

66 Doc: not wanting to minimise your concerns as well yeah

67  (1.0)

68 Dad: Yeah well you know Mary’s a nurse and midwife as well so she’s

69   pretty ah- pretty strict on all of us for now (.) we’re uh wash

70   hands and obviously use the ha(h)nd sanitiser and things like that

71 Doc: [Yeah]

72 Dad: [And ] we haven’t taken our- either of the kids out u::m (.)

73   anywhere where they shouldn’t be we’ve- we’ve pretty much done the

74   right thing the whole way along so

75 Doc: Yeah.

76 Dad: ah better safe than sorry.

The doctor’s recommendation at lines 1-2 involves standard advice related to giving the child *Nurofen®*. As seen in Fragment 1 above, recommendations to give paracetamol or ibuprofen for pain relief is a common practice in paediatric palliative care consultations. They are often used as pain relief for mild to moderate pain and are typically accepted by parents without further discussion. In this instance, however, this standard recommendation leads to a different response from the father. The father provides some initial agreement (‘yeah yeah’, line 4) and then provides a ‘but’ conjunction (‘but we’ve had some concerns with coronavirus’). The conjunctive ‘but’ here acts as a pivot in the conversation: rather than just responding with the expected acceptance of the recommendation, the father raises a new topic (coronavirus) and presents a new concern.

This is the first time that coronavirus is mentioned in the consultation; there had been no lead up to the shift in conversation from the father at this point. The father’s concern is expanded in lines 6-9 when he explains that he has heard that *Nurofen®* is not the best medication to use ‘at the moment’, that is, during the coronavirus pandemic. Here, the father refers to media reports at the time that suggested that ibuprofen might aggravate the symptoms of coronavirus (this link was subsequently debunked).^36,37^ Even though the child did not have coronavirus, the father expressed concern about using *Nurofen®* during the pandemic. From line 10, across an extended sequence, the doctor responds by providing both information and reassurance to the father. The father accepts this response from the doctor (‘Yep that’s fine’ line 43, ‘That’s good to know’, line 51), and finishes with an idiomatic expression (‘better safe than sorry’, line 76), which works to close this topic.^38,39^ This fragment shows how, during the pandemic, a relatively simple recommendation to give a child mild pain relief led to a parental concern being raised, and a need for the doctor to provide up-to-date information about COVID-19 and reassurance to the parent in response.

A second serious concern raised by parents within consultations recorded during the pandemic was attending the hospital for care. Prior to the pandemic, families often attended the hospital for regular consultations, including travelling long distances from remote towns to see clinicians face-to-face. As the next fragment shows, however, some parents expressed caution in visiting the hospital during the pandemic, even when concerned about their child’s immediate health problems. In this next fragment, a telehealth consultation, the parents are concerned about their son’s breathing. They had told the doctor previously that they did not want to visit the hospital due to the risk of COVID-19.

**(6) [S1_F25_E01_2020-04-30 07:48]**

01 Doc:  o:kay. (1.2) .hh AND UM .hh you’re- YOU:’re- (.) YOU’re not

02         keen to come up to the hospital at the moment are you?

03        (1.3)

04 Dad:  n↑a:h.

05        (0.3)

06 Doc:  mhm.

07 Dad:  er I- I like to visit you to: say gidday and all ↑that [but ah]

08 Doc:                                                              [mm    ]

09        (0.5)

10 Dad:  I’m very conc- <I:’m overly cautious> with this COVID nineteen

11        thing (0.7) er if I: get it (.) Ryan gets it (.) and we’re a

12  go:ner.

13 Doc:  mhm=

14 Dad:  =y’know,

15 Doc:  mhm

16        (0.8)

17 Dad:  erm (.) and ↑I KNOW the: hospital’s got a (.) clean slate at

18        the moment >but look at that nursing home down in ↑Sydney. .hh

19        wh- where there’s casual workers you’ve got a ↑risk.

20        (0.3)

21 Doc:  mhm.

22        (0.7)

23 Dad:  and you’ve got a risk in the hospital.

24        (1.0)

25 Doc:  o:kay.

In this Fragment 6, at lines 1-2, the doctor asks the parents about visiting the hospital. The question is framed as a negative declarative, displaying the doctor’s prior knowledge that the parents are ‘not keen’ to visit the hospital despite their current concerns about their son’s dystonia and breathing. Within his question, the doctor refers to the COVID-19 pandemic through to the temporal reference ‘at the moment’. The father provides a straight no-response: ‘nah’ (line 3). He then expands this response, explicitly raising COVID-19 as the justification for his unwillingness to visit the hospital. The father’s turn contains an intensifier (‘overly cautious’, line 10) and the use of the idiomatic expression ‘we’re a goner’ to make reference to the risk of the child’s death if he caught the coronavirus (lines 11-12). The father thus uses accentuated and emotive language to emphasise his perceived risk of visiting the hospital in person. This risk appears to be a greater concern to the parents than continuing to care for the child’s health concern at home. This fragment shows another example of how serious concerns about the pandemic were raised by parents during consultations while discussing routine aspects of the child’s ongoing care.

A third form of talk about the COVID-19 pandemic became relevant in interactions when talking about issues related to the families’ lifestyle, such as school (Fragment 7) and work (Fragment 8).

**(7) [S1_F23_E01_2020-04-09 16:57]**

01 Doc: ◦okay◦ (1.3) u:m okay (.) so um (.) so that’s a good point .hh

02   and um .hh wha- what schoo:l does Sammy go to?

03   (1.3)

04 Dad: tsk um [Rickshaw State]

05 Pat:   [Rickshaw State] School

06   (1.3) ((doc typing))

07 Doc: .hh and what grade are you in Sammy?

08   (1.1)

09 Pat:  fi:ve.

10 Doc: ◦okay◦ (0.8) AND UM (0.3) did you um- (.) >how many (0.3) when

11   did you sort of stop going to schoo:l ah did you:: (.) like um

12   (0.8) a:h (0.3) two weeks ago:, or a month ago:,

13   (1.2)

14 Pat: tsk o:h. I: ((shaking head))

15   (0.4)

16 Dad: three weeks.

17 Doc: [okay yeah ]

18 Pat: [(yeah a month)] ago.

19 Doc: ◦yea:h.◦

20   (0.3)

21 Dad: basically u::m (.) y’know before the lockdowns happened we were

22   about a- a week before tha:t (.) sort of started (1.0) ((doc

23   typing)) we- we (.) >y’know we wanted to be proactive< once

24   again (.) with this

25   (.)

26 Doc: yeah

27 Dad: didn’t want to take any chances

This fragment begins with the same question from the doctor as seen in Fragment 2 above from the pre-pandemic consultations: asking what school the child attends. After receiving information from the child and father about the child’s school and grade level, the doctor asks another question that shifts the conversation to the relevance of the pandemic. At the time of this consultation, schools were providing home learning and only children of essential workers were physically attending school. In this community-wide context of most children not attending school, asking the child about his school raises the relevance of the temporary change to school routine that the child would be experiencing.

The doctor’s next question, across lines 10-12, orients to this relevance by asking how long the child has been home from school. Both the child and father reply with the number of weeks the child has been home, and the father then expands by explaining that they were ‘proactive’ and the child started staying home from school around a week before the official school closures (‘before the lockdowns happened’, line 21). The father’s response concluded with an idiomatic expression that they ‘didn’t want to take any chances’.^38,39^ With this response, the father displays to the doctor that he took the child’s isolation from the community seriously and took precautions beyond the standard recommendations. Again, this fragment demonstrates how a routine question about the child’s school, asked during the pandemic period, leads to a shift in the conversation to pandemic-related lifestyle changes including home-schooling and the father displaying he was taking isolation of the child seriously.

In Fragment 8, below, the child’s father has already spoken about the child’s mother (Mary) working as a nurse (discussed in Fragment 5 above). At the beginning, the doctor enquires about the father’s own work responsibilities.

**(8) [S1_F23_E01_2020-04-09 27:57]**

01 Doc: um (.) .hh Henry um do you um (◦eh◦) (0.3) like with Ma:ry

02   worki:ng .hh are you: >sort of< working as well or you’re

03   mainly more home based? or

04   (1.3)

05 Dad: I work from home at the mo:ment, ye[a:h ] which is ah .hh

06 Doc:      [okay]

07 Dad: >not doing a lot of work< I’m in the u:m (.) tsk in the gaming

08   industry so:: dealing with casinos and clubs an- and pubs and

09   that sort of thing so I think .hh everything’s shut do:wn at

10   the moment it’s very quiet,

11   (0.7)

12 Doc: yea:h (2.5) ◦and um◦ so you s- can still do a little bit of

13   work (.) remotely hhh

14   (0.8)

15 Dad: yeah I work from home ni- l-=

16 Doc: =yeah=

17 Dad: =ni:nety per cent of the ti:me and then I travel (.) a:h >for

18   the< [rest]

19 Doc:   [yeah]

20 Dad: so: .hh when (.) before coronavirus >I was< on the road

21   probably about w- (.) one week out of every ↑four

22   (0.3)

23 Doc: yea:h.

24 Dad: most of my work’s over (in Asia) so:

25   (0.3)

26 Doc: yeah. okay, (1.7) and um tsk h- okay yeah >and I guess that’s

27   probably a:h has that had sort of a financial impact on you

28   guys: um

29   (2.0)

30 Dad: yea:h I- I’ve- I was pretty lucky (when) (0.3) I’ve take a

31   twenty per cent pay cut (.) to get us through this period which

32   is (0.4) you know not too bad it’s better than the Job Keeper

33   payment that’s for sure.

34 Doc: yeah okay yeah well that’s a relief yeah (0.3) and um .hh

35   Mary’s work I guess um (0.3) (w-/eh) fortunately they’ll be:

36   wanting her to continue in that role won’t they so ◦yeah◦ .hh

37 Dad: yeah yeah [we’ve-] we’re good from that perspective we’re-

38 Doc:    [yeah ]

39 Dad: we’re the lucky ones.

The doctor’s question at lines 1-3 is a routine question often observed in the paediatric palliative care consultations and can be compared to the similar question seen in Fragment 3 above. The father initially responds that he works ‘from home at the moment’ (line 5). With this initial response, the father already makes an apparent reference to the pandemic period, marking his current home-based work routine as being different to usual. In his expanded response across lines 7-10, the father explains he is in the ‘gaming industry’ and makes further reference to the pandemic in stating that ‘everything’s shut down at the moment it’s very quiet’. In a similar way to the other fragments, the father’s response to a routine question shifts the conversation to the changed routine due to COVID-19. Further in the sequence, the father explains that, ‘before coronavirus,’ he travelled one week in four for his work (lines 20-21). With travel bans in place at the time, the father’s response here suggests that his job probably has been impacted significantly during the pandemic.

What was a routine question has led now to some sensitive information-sharing by the father related to the impact of the pandemic on the family. The doctor orients to this sensitivity by making an inference: ‘I guess that’s probably ah has that had sort of a financial impact on you guys’ (lines 26-28). The father confirms the financial impact and further shares that he had to take a 20% reduction in salary during this time (lines 30-33). He follows on with a positive stance, that this was better than losing his job (as many Australians did) and needing to rely on Government subsidy payments. The doctor provides an empathic receipt (‘that’s a relief’, line 34) and adds that at least his wife’s work (as a nurse) will continue. The father aligns with the doctor, using the idiomatic expression ‘we’re the lucky ones’ to close down the sequence^38,39^ (line 39). Again, here, a routine question from the doctor quickly shifts into talk about the pandemic; in this case leading to sensitive talk in relation to the impact of the pandemic on the parent’s work and finances, requiring an empathic response from the doctor.

The final example shows how talk about the pandemic led to non-serious talk and laughter in some consultations. The fragment comes from an inpatient consult where the child is lying in bed watching a TV show on a digital tablet.

**(9) [S1_F24_E02_2020-04-17 00:29]**

01 Doc: have u:m (0.3) >so that-< which tha:t’s which ↑one Sesame

02   Street. (.) or=

03 Mum: =Yep

04 Doc: [Okay ]

05 Mum: [It’s lit]erally the only show she watches.

06 Doc: Okay yea:h.

07 Mum: Yep.

08   (0.7)

09 Doc: >actually I ↑see y-< I sa:w Sesame Street have done a thing on:

10   (.) COVID  nine↑teen so[: ]

11 Nur:     [◦oh hh◦]

12 Doc: yea:h. so:=

13 Mum: =heh. (.) [£there’s a] me:me (.) that says£=

14 Doc:   [yea:h. ]

15 Doc: =yeah.

16   (0.2)

17 Mum: £Sesame Street i(h)s brou(h)ght to you by the letter (.) ↑C:

18   (.) and the n:umber nineteen£

19 Nur: Heh [heh heh] heh heh ]

20 Doc:  [Heh heh]

21 Mum:  [Hah hah hah hah hah] hah hah hah=

22 Nur: =hh=

23 Mum: =.hh hh .hh

24   (0.2)

25 Doc: ◦yeah◦

In a similar way to Fragment 4 (a pre-pandemic consultation), the doctor (line 2) enquires as to whether the TV show the child is currently watching is Sesame Street but leaving incomplete his turn for other possible programs. The mother confirms and adds ‘it’s literally the only show she watches’ (line 5), which is similar to the mother’s response in Fragment 4. Subsequently, the doctor makes reference to COVID-19 in relation to the show: ‘Sesame Street have done a thing on COVID-19’ (lines 8-9).^40^ The mother responds by relaying the details of a Sesame Street meme she had seen in relation to COVID-19 (lines 12, 14-15). Her turn displays the laughable nature of the meme, indicated by smile voice and interpolated laugh particles. The mother, doctor and nurse all join in laughter in response (lines 16-18) and this shared laughter closes down the sequence.^41,42^ Similar to the other fragments, a commonly observed question from the doctor leads to talk about the COVID-19 pandemic. In this instance, the COVID-19 talk leads to appreciation of a meme with shared laughter.

## Discussion

Guidelines released for inpatient and outpatient care during the COVID-19 pandemic provide recommendations for framing a *specific* conversation about COVID-19 with patients.^20–23,26^ While these guidelines are valuable, this study has shown that talk about the COVID-19 pandemic was pervasively raised by both parents (45%) and clinicians (55%) *throughout* actual consultations. Routine questions and recommendations from the doctor often inadvertently raised the relevance of the COVID-19 pandemic and the associated changes to care and lifestyle.

It appears helpful to have direct and specific questions to ask about COVID-19 (e.g. ‘What have you been thinking about COVID and your situation?’).^23^ At the same time, parents and patients often raised the topic themselves or in response to indirect questioning such as a check-in type question (‘How are you doing with all of this?’).^23^ The holistic and comprehensive nature of a paediatric palliative care consultation can also facilitate conversation on a variety of topics ranging from school to family functioning.^13,14^ More serious health related conversations also occurred around pain and symptom management, and how to manage deterioration of the child if this were to occur. The importance of maintaining emotional support, empathy and compassion during such sensitive conversations is critical. As shown in the reported data (e.g., Fragment 6), families who might otherwise physically attend consultations shifted to telehealth consultations during the pandemic. Virtual and telehealth technologies present extra challenges to providing psychosocial support, but do not preclude it.^43–45^

### Strengths and limitations

This is the first study to directly analyse real-life, video-recorded paediatric palliative care consultations prior to, during, and immediately following the initial peak of the COVID-19 pandemic. It provides insight into how the communication between clinicians and families changed as a result of the pandemic in these consultations. It adds to the small, and growing, body of research examining naturally-occurring communication within paediatric palliative care.^33,34^ A limitation of the study is that data were collected from one hospital in Australia. Another study limitation is that the diagnoses in this study are not totally representative of the patients in this service. Thirty-five percent of patients usually seen by this service have an oncologic diagnosis, not present in the current study.^46,47^ Future research might seek to include a representative group of diagnoses, to collect data at additional sites and locations, as well as during future waves of the pandemic (e.g., one State in Australia is currently experiencing a second wave of COVID-19 in July 2020, including cases within a Children’s hospital).

### Practice implications

An awareness of the pervasiveness of both serious and non-serious talk about the COVID-19 pandemic within standard paediatric palliative care consultations during the pandemic can encourage clinicians to be prepared and flexible in how they respond to patients. In some instances, the pandemic influenced families’ decision-making about the way their child received care from the palliative care service. For example, Fragment 6 showed that a family was hesitant to come to hospital for inpatient care. In other consultations, some concerns may be raised by families that require reassurance in response from clinicians. Other topics of conversation may require a sensitive acknowledgement of the additional life stressors that families can confront during pandemics (e.g., home schooling, financial stress due to loss of reliable employment). Pandemic topics may be raised also when discussing other psychosocial or lifestyle issues, such as education (e.g., remote school learning) or culture (e.g., television programs). Some of these conversations may involve more light-hearted discussion around the changes to everyday routines. Given the holistic purpose of paediatric palliative care in providing both medical and supportive care to families,^12–14^ this service is in an ideal position to listen to and meet families’ concerns to talk about pandemic-related matters that may potentially affect their child’s health outcomes and family’s everyday practices.

Guidelines based on real-life conversations, rather than on imagined scripts of conversations, are more likely to show the diverse range of talk about the pandemic within clinical consultations.^35^ It is unlikely that script writers could ever imagine the diversity of content, far broader than health concerns, that may surface within consultations during pandemics. Future guidelines might consider the pervasive and varied ways that conversations about a pandemic are raised within and across routine consultations to prepare clinicians for the flexible ways that they may need to respond to patients and families during such challenging times.

## Appendix 1: Jeffersonian transcription system

This list represents the most widely-used transcription symbols used in this study. For a more comprehensive list, see Jefferson.^29^

(.)   Micro-pause – less than a tenth of a second

(0.2), (2.6)   Examples of timed pauses

↑word    Onset of noticeable pitch rise

↓word   Onset of noticeable pitch fall

A: word [word   Square brackets aligned across adjacent lines denote the start of

B: [word   overlapping talk.

.   Falling vocal pitch

?   Rising vocal pitch

.hhh   n-breath

hhh   Out-breath

wo(h)rd   Within-speech aspirations

wor-   A sharp cut-off

wo:rd   Colons show that the speaker has stretched the preceding sound

(words)   A guess at what might have been said if unclear

(  )   Unclear talk

A: word=   The equals sign shows that there is no discernible pause

B: =word   between two speakers’ turns

word   Vocal emphasis

WORD   Talk pronounced loudly in comparison with surrounding talk

°word°   Talk between ‘degree signs’ is quieter than surrounding talk

>word word<    Talk between inward arrows is delivered faster than surrounding talk

<word word>   Talk between outward arrows is delivered slower than surrounding talk

((*sniff*))   Transcriber’s effort at representing something difficult, or impossible, to write phonetically

£word£   Words spoken with smiley voice

## References

[1] Mehta NS Mytton OT Mullins EWS, et al SARS-CoV-2 (COVID-19): what do we know about children? A systematic review. Clin Infect Dis. Epub ahead of print 12 5 2020 DOI: 10.1093/cid/ciaa556. PMC7239259 32392337

[2] Ludvigsson JF Systematic review of COVID-19 in children shows milder cases and a better prognosis than adults. Acta Paediatr 2020; 109: 1088–1095. 32202343 10.1111/apa.15270 PMC7228328

[3] Zimmermann P Curtis N Coronavirus infections in children including COVID-19: an overview of the epidemiology, clinical features, diagnosis, treatment and prevention options in children. Pediatr Infect Dis J 2020; 39: 355–368. 32310621 10.1097/INF.0000000000002660 PMC7158880

[4] Chiotos K Hayes M Kimberlin DW, et al Multicenter initial guidance on use of antivirals for children with coronavirus disease 2019/severe acute respiratory syndrome coronavirus 2. J Pediat Inf Dis Soc. in press. DOI: 10.1093/jpids/piaa045. PMC7188128 32318706

[5] Sinha IP Harwood R Semple MG, et al COVID-19 infection in children. Lancet Respir Med 2020; 8: 446–447. 32224304 10.1016/S2213-2600(20)30152-1 PMC7154504

[6] Jeng M-J. COVID-19 in children: current status. J Chin Med Assoc. in press. DOI: 10.1097/jcma.0000000000000323. PMC7199766 32502117

[7] Dayal D We urgently need guidelines for managing COVID-19 in children with comorbidities. Acta Paediatr. in press. DOI: 10.1111/apa.15304. PMC7262043 32279351

[8] Weaver MS Wiener L Applying palliative care principles to communicate with children about COVID-19. J Pain Symptom Manag. in press. DOI: 10.1016/j.jpainsymman.2020.03.020. PMC7270779 32240751

[9] Shekerdemian LS Mahmood NR Wolfe KK, et al Characteristics and outcomes of children with coronavirus disease 2019 (COVID-19) infection admitted to US and Canadian pediatric intensive care units. JAMA Pediatr. Epub ahead of print 11 5 2020 DOI: 10.1001/jamapediatrics.2020.1948. PMC7489842 32392288

[10] CDC COVID-19 Response Team. Coronavirus disease 2019 in children - United States, February 12-April 2, 2020. MMWR 2020; 69: 422–426. 32271728 10.15585/mmwr.mm6914e4 PMC7147903

[11] Jansen M Irving H Gillam L, et al Ethical considerations for paediatrics during the COVID -19 pandemic: a discussion paper from the Australian Paediatric Clinical Ethics Collaboration. J Paediatr Child Health 2020; 56: 847–851. 32471008 10.1111/jpc.14946 PMC7300784

[12] Muckaden M Dighe M Balaji PD, et al Paediatric palliative care: theory to practice. Indian J Palliat Care 2011; 17(Suppl): S52–S60. 21811373 10.4103/0973-1075.76244 PMC3140086

[13] Hartley G Berger Z Maynard L The development and evaluation of a holistic needs assessment within children’s palliative care. Int J Palliat Nurs 2016; 22: 236–242. 27233011 10.12968/ijpn.2016.22.5.236

[14] Bradford N Herbert A Mott C, et al Components and principles of a pediatric palliative care consultation: results of a Delphi study. J Palliat Med 2014; 17: 1206–1213. 25006759 10.1089/jpm.2014.0121

[15] Jones BL Contro N Koch KD The duty of the physician to care for the family in pediatric palliative care: context, communication, and caring. 2014; 133: S8–S15. 10.1542/peds.2013-3608C 24488541

[16] Boucher S Atout M McNamara-Goodger K Communication with children and their families. In: Downing J (ed.) Children’s palliative care: an international case-based manual. Cham: Springer Nature Switzerland AG, 2020, pp. 51–63.

[17] Murray CD McDonald C Atkin H The communication experiences of patients with palliative care needs: a systematic review and meta-synthesis of qualitative findings. Palliat Support Care 2015; 13: 369–383. 24784479 10.1017/S1478951514000455

[18] Spicer J Chamberlain C Papa S Provision of cancer care during the COVID-19 pandemic. Nat Rev Clin Oncol 2020; 17: 329–331. 32296166 10.1038/s41571-020-0370-6 PMC7156894

[19] Mehta AK Smith TJ Palliative care for patients with cancer in the COVID-19 era. JAMA Oncol. Epub ahead of print 7 5 2020 DOI: 10.1001/jamaoncol.2020.1938. 32379855

[20] Calton B Abedini N Fratkin M Telemedicine in the time of coronavirus. J Pain Symptom Manag 2020; 60(1): e12–e14. 10.1016/j.jpainsymman.2020.03.019 PMC7271287 32240756

[21] Back A Tulsky JA Arnold RM Communication skills in the age of COVID-19. Ann Intern Med. Epub ahead of print 2 6 2020 DOI: 10.7326/M20-1376. PMC7143152 32240282

[22] Finset A Bosworth H Butow P, et al Effective health communication – a key factor in fighting the COVID-19 pandemic. Patient Educ Couns 2020; 103: 873–876. 32336348 10.1016/j.pec.2020.03.027 PMC7180027

[23] Bowman BA Back AL Esch AE, et al Crisis symptom management and patient communication protocols are important tools for all clinicians responding to COVID-19. J Pain Symptom Manag 2020; 60(2): e98–e100. 10.1016/j.jpainsymman.2020.03.028 PMC7141479 32276102

[24] VitalTalk. COVID ready communication playbook, https://www.vitaltalk.org/guides/covid-19-communication-skills/ (2020, accessed 1 June 2020).

[25] Kelemen A Altilio T Leff V Specific phrases & word choices that can be helpful when dealing with COVID19, https://www.professionalchaplains.org/Files/resources/COVIDLanguageGuideFinal.pdf (2020, accessed 1 June 2020).

[26] Ariandne Labs. Serious illness care program COVID-19 response toolkit, https://covid19.ariadnelabs.org/serious-illness-care-program-covid-19-response-toolkit/#inpatient-resources (2020, accessed 1 June 2020).

[27] Cucinotta D Vanelli M WHO declares COVID-19 a pandemic. Acta Biomedica 2020; 91: 157–160. 32191675 10.23750/abm.v91i1.9397 PMC7569573

[28] DOH. COVID-19, Australia: epidemiology report 16. In: Intelligence CD (ed.). Canberra, ACT: Australian Government Department of Health, 2020, pp. 1–31.

[29] Jefferson G Glossary of transcript symbols with an introduction. In: Lerner G (ed.) Conversation analysis: studies from the first generation. Amsterdam: John Benjamins Publishing Company, 2004, pp. 13–31.

[30] Sidnell J Basic conversation analytic methods. In: Sidnell J Stivers T (eds) The handbook of conversation analysis. Chichester: Blackwell Publishing Ltd, 2013, pp. 77–99.

[31] Drew P Chatwin J Collins S Conversation analysis: a method for research into interactions between patients and health-care professionals. Health Expect 2001; 4: 58–70. 11286600 10.1046/j.1369-6513.2001.00125.x PMC5060048

[32] Heritage J Maynard DW Problems and prospects in the study of physician-patient interaction: 30 years of research. Annu Rev Sociol 2006; 32: 351–374.

[33] Ekberg S Danby S Herbert A, et al Affording opportunities to discuss deterioration in paediatric palliative care consultations: a conversation analytic study. BMJ Support Palliat Care 2017; 10(2): 1–9. 10.1136/bmjspcare-2016-001130 28270396

[34] Ekberg S Danby S Rendle-Short J, et al Discussing death: making end of life implicit or explicit in paediatric palliative care consultations. Patient Educ Counsel 2019; 102: 198–206. 10.1016/j.pec.2018.08.014 30236971

[35] Sacks H Notes on methodology. In: Atkinson JM Heritage J (eds) Structures of social action: studies in conversation analysis. Cambridge: Cambridge University Press, 1984, pp. 21–27.

[36] McKenna M The ibuprofen debate reveals the danger of Covid-19 rumors. New York: Wired, 2020.

[37] SBS. WHO warns against use of ibuprofen for coronavirus symptoms, https://www.sbs.com.au/news/who-warns-against-use-of-ibuprofen-for-coronavirus-symptoms (2020, accessed 3 June 2020).

[38] Antaki C Mental-health practitioners’ use of idiomatic expressions in summarising clients’ accounts. J Pragmat 2007; 39: 527–541.

[39] Drew P Holt E Figures of speech: figurative expressions and the management of topic transition in conversation. Lang Soc 1998; 27: 495–522.

[40] Sesame Street. Caring for Each Other, https://www.sesamestreet.org/caring (2020, accessed 6 May 2020).

[41] Holt E The last laugh: Shared laughter and topic termination. J Pragmat 2010; 42: 1513–1525.

[42] Holt E Reporting and reacting: concurrent responses to reported speech. Res Lang Soc Interac 2000; 33: 425–454.

[43] Bradford N Armfield N Young J, et al Principles of a paediatric palliative care consultation can be achieved with home telemedicine. J Telemed Telecare 2014; 20: 350–364. 10.1177/1357633X14552370 25399995

[44] Ellis K Lindley LC A virtual children’s hospice in response to COVID-19: the Scottish experience. J Pain Symptom Manag 2020; S0885-3924(20)30387-0. DOI: 10.1016/j.jpainsymman.2020.05.011. PMC7228698 32416232

[45] Solli H Hvalvik S Nurses striving to provide caregiver with excellent support and care at a distance: a qualitative study. BMC Health Serv Res 2019; 19: 893. 31771566 10.1186/s12913-019-4740-7 PMC6880571

[46] Herbert A Bradford N Donovan L, et al Development of a state-wide pediatric palliative care service in Australia: referral and outcomes over two years. J Palliat Med 2014; 17: 288–295. 24528125 10.1089/jpm.2013.0400

[47] Nolte-Buchholtz S Zernikow B Wager J Pediatric patients receiving specialized palliative home care according to German law: a prospective multicenter cohort study. Children 2018; 5: 66. 10.3390/children5060066 PMC6028915 29857504

